# Antidotes in Strychnine, Resorcin and Picrotoxine

**Published:** 1888-01

**Authors:** 


ANTIDOTES TO STRYCHNINE, RESORCIN, AND PICROTOXINE.—Professor Aurep has proven experimentally that urethan possesses properties antagonistic to the convulsive drugs, strychnine, etc., and believes that urethan may be employed in cases of poisoning, by these substances. It is superior, for this purpose, to hydrate of chloral; it is less dangerous, and may be administered in large doses with perfect safety. The author concludes that, in the case of man, it is necessary to administer it in doses of four to six grains, as an antidote to the poisons above mentioned.

E. Kock states that butyl-chloral hydrate is useless as an antidote in cases of strychnine-poisoning; in picrotoxine-poisoning, it fails to overcome three times the minimum fatal dose, behaving, in this respect, like chloral-hydrate. Picrotoxine may be successfully used as an antidote to the narcotic effects of butyl-chloral hydrate and chloral-hydrate. According to A. Bockal, paraldehyde is a powerful antidote to strychnine. Ten times the fatal dose of strychnine may be safely administered to dogs that have previously received paraldehyde. Strychnine is not, however, an antidote to paraldehyde.

